# Diffuse gliomas: insights into clinical and histopathological features and survival rates from two centers in a middle-income country

**DOI:** 10.3389/fonc.2025.1529456

**Published:** 2025-07-04

**Authors:** Edgar G. Ordóñez-Rubiano, Cristian Siabato, Nicolás Rincón-Arias, Paula A. Pulido, Hebert D. Pimienta-Redondo, Sebastián Espinosa-Gaona, Santiago Useche-Diosa, María Andrea Moreno, Hernando A. Cifuentes-Lobelo, Oscar F. Zorro-Guio, Javier G. Patiño-Gómez, Alba Lucía Cómbita, César Payán-Gómez, Luz D. Gutierrez-Castañeda, Alexandra Ramos-Márquez, Diego F. Gómez, Oscar Mendoza, Matias Baldoncini, Rafael Parra-Medina

**Affiliations:** ^1^ School of Medicine, Universidad Nacional de Colombia., Bogotá, Colombia; ^2^ Department of Neurosurgery, Fundación Universitaria de Ciencias de la Salud, Hospital de San José – Sociedad de Cirugía de Bogotá, Bogotá, Colombia; ^3^ Department of Neurosurgery, Hospital Universitario Fundación Santa Fe de Bogotá, Bogotá, Colombia; ^4^ Group of Basic Health Sciences (CBS), Fundación Universitaria de Ciencias de la Salud, Bogotá, Colombia; ^5^ Department of Neurosurgery, Fundación Universitaria de Ciencias de la Salud, Hospital Infantil Universitario de San José, Bogotá, Colombia; ^6^ Universidad Nacional de Colombia, Facultad de Medicina, Departamento de Microbiología, Instituto Nacional de Cancerología, Grupo de Investigación Traslacional en Oncología., Bogotá, Colombia; ^7^ Dirección Académica, Universidad Nacional de Colombia, Sede de La Paz, Cesar, Colombia; ^8^ Research Institute, Fundación Universitaria de Ciencias de la Salud, Bogotá, Colombia; ^9^ School of Medicine, Universidad de los Andes, Bogotá, Colombia; ^10^ Department of Pathology, Fundación Universitaria de Ciencias de la Salud, Hospital de San José, Bogotá, Colombia; ^11^ Microsurgical Neuroanatomy Laboratory Director, Universidad de Buenos Aires, Buenos Aires, Argentina; ^12^ Departament of Neurosurgery, Hospital San Fernando, Buenos Aires, Argentina; ^13^ Departament of Pathology, Instituto Nacional de Cancerología Bogotá, Bogotá, Colombia

**Keywords:** glioma, astrocytoma, oligodendroglioma, glioblastoma, Colombia, survival

## Abstract

**Introduction:**

Gliomas are believed to arise from neuroglial stem cells and are histologically classified based on morphological similarities to normal neuroglial cells. This study aims to describe the clinical, histopathological, and demographic features of glioma patients treated in two reference centers in Colombia.

**Methods:**

This descriptive cross-sectional study included all patients with a histologically confirmed glioma treated at two institutions in Bogotá, Colombia, between January 2015 and December 2023. 272 patients with diffuse gliomas were included, and data were collected on sociodemographic characteristics, clinical presentation, histopathologic diagnosis, immunohistochemical markers, extent of resection, functionality, complications, and survival.

**Results:**

Amongst all cases, 36.00% were glioblastomas, 14.70% oligodendrogliomas, and 12.10% astrocytomas. 49.10% of patients were females, average age was 48.8 ± 21.0 years. While in the frontal lobe, most glioblastomas (38.95%) and oligodendrogliomas (47.50%) were found, astrocytomas were more frequent in the insula (27.27%). The average follow-up was 11.8 ± 16.0 months. Near-total resection was achieved in 40.10% of patients, followed by subtotal resection (37.00%), gross-total resection (11.45%), and intentional biopsy (11.45%). 31.25% of patients had new-onset motor deficits, and only 3% persisted after the 3-month follow-up. Overall survival was higher in females (males: 28.57% vs. females: 55.00%) (p = 0.0013). The 2-year overall survival for glioblastoma was 21%, 5-year for glioma, NOS 38%, for astrocytoma 15%, and 8-year for oligodendroglioma 5% (p < 0.0001).

**Conclusions:**

We present the largest study to date of diffuse glioma in Colombia’s population. Clinical findings and overall survival trends are similar to those reported worldwide, however, further molecular analysis is needed for adequate diagnosis and classification

## 1 Introduction

According to the Global Cancer Observatory (GLOBOCAN) the incidence of central nervous system (CNS) malignant tumors was 3.1-3.9 per 100,000 in 2022 (1), with a prevalence of 10.1 per 100,000 people worldwide according to the 2020 report (2). Gliomas account for almost 30% of all primary brain tumors and 80% of all malignant tumors; they are responsible for the majority of deaths from primary brain tumors (3, 4). Gliomas are believed to arise from neuroglial stem cells and are histologically classified into astrocytomas, oligodendrogliomas, and glioblastomas (GBMs), based on morphological similarities to neuroglial cells found in healthy brains. Further classification is performed according to the location of the tumor and the anaplastic features (mitotic activity, microvascular proliferation, and necrosis). The absence or presence of anaplastic features is used to assign grades of malignancy from I to IV according to the fourth edition of the World Health Organization (WHO) classification of tumors of the CNS (WHOCNS4) (5–7). According to the WHOCNS4 (5) gliomas include gliomas of various grades [pilocytic astrocytoma (grade I), diffuse astrocytoma (grade II), anaplastic astrocytoma (grade III), GBM (grade IV)], oligodendrogliomas (grade II and III), and the group controversial mixed oligoastrocytomas (grade II and III) (4, 5). Survival rates vary depending on histology, with pilocytic astrocytoma patients experiencing 10-year survival rates of over 90%, while in GBM, a mere 6,8% of patients reach the 5-year survival mark (8, 9).

In recent years, there has been significant progress in the molecular analysis of gliomas. These advances have resulted in improved classification systems based on mutational profiles (10). However, the costs related to molecular profiling for diagnosing and classifying gliomas remain elevated, and clinical use in lower-to-middle-income countries (LMICs) is limited. Additionally, in most LMICs like Colombia, there is scarce national information systems data due to several limitations, including underreporting and partial general coverage, posing many challenges for evaluating the epidemiology of gliomas. Furthermore, the study of different pathologies, including gliomas is particularly challenging given that Colombia’s population has a varied ethnicity and genetic ancestry, including Afro-descendant, Indigenous, Mulatto, Black, Palenquero, Raizal, and Rom people (11). In Colombia, gliomas represent ~30% of intracranial tumors (12, 13). The most common primary malignant brain tumor in Colombia is GBM (14). The estimated 3-year overall survival (OS) in Colombia of patients with GBM is 12% (15). To our knowledge, this is the largest series of patients with diffuse gliomas treated in Colombia. This study aims to describe the clinical, histopathological, and demographic features of diffuse glioma patients treated in two reference centers in Colombia.

## 2 Materials and methods

**Figure 1:**
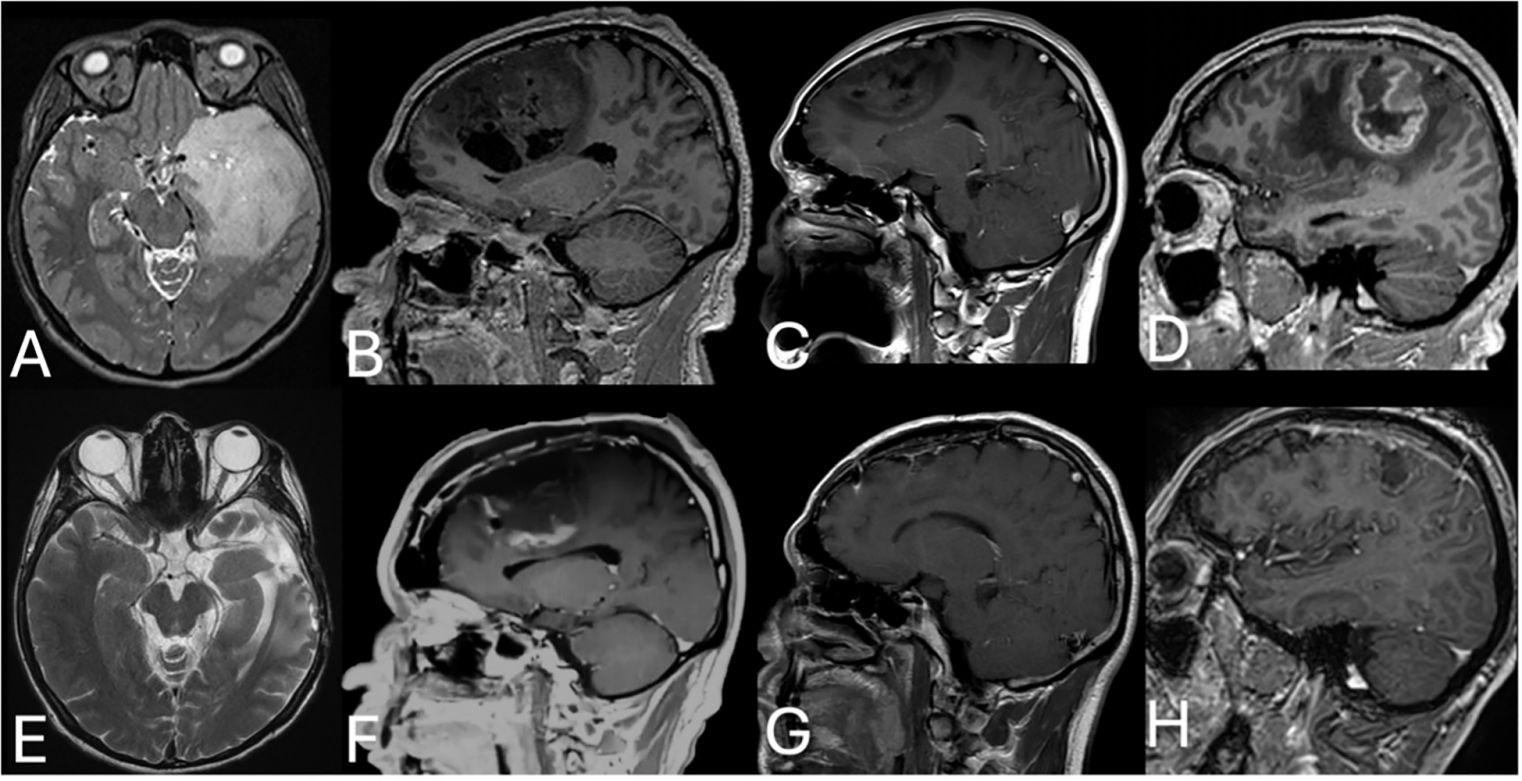
Pre- and postoperative MRI of diffuse gliomas. **(A)** Pre- and **(E)** postoperative MRI of a left temporal grade II astrocytoma. **(B)** Pre- and **(F)** postoperative enhanced MRI of a right frontal grade III oligodendroglioma. **(C)** Pre- and **(G)** postoperative enhanced MRI of a left frontal low-grade glioma (glioma, NOS). **(D)** Pre- and **(H)** postoperative enhanced MRI of a right perirolandic GBM.

**Figure 2:**
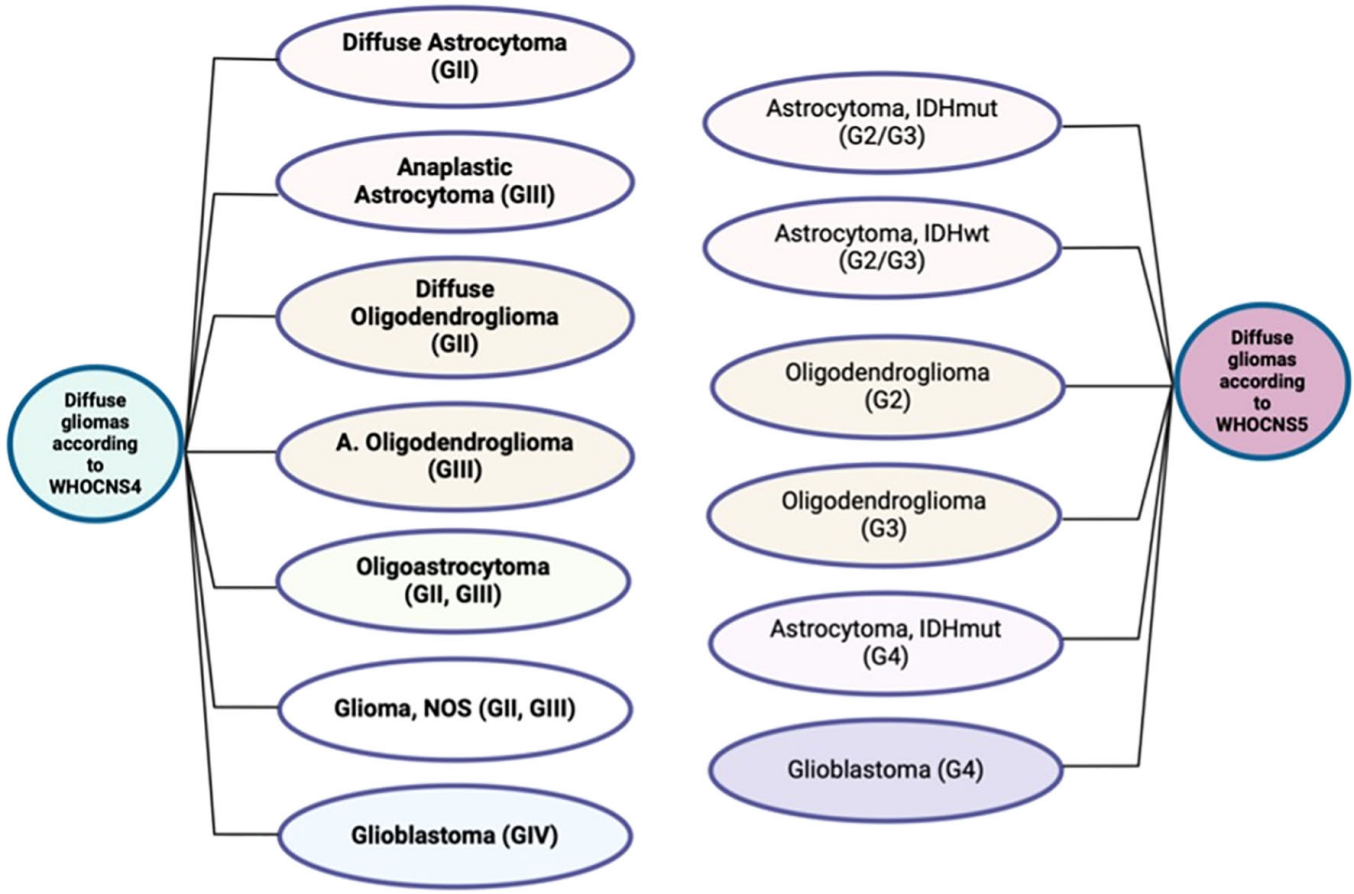
Diffuse gliomas according to the fourth and fifth editions of the World Health Organization classifications of central nervous system tumors. *WHOCNS4 = fourth edition of the World Health Organization Classification of Tumors of the Central Nervous System, WHOCNS5 = fifth edition of the World Health Organization Classification of Tumors of the Central Nervous System, IDHmut = isocitrate dehydrogenase 1 mutant, IDHwt = isocitrate dehydrogenase 1 wildtype, A. Oligodendroglioma: anaplastic oligodendroglioma.* *Grade I gliomas are not included in this illustration as the WHOCNS5 considers them as a different entity.

This is a descriptive cross-sectional study that included all the patients with a histologically verified diffuse glioma who were treated at Hospital de San José – Sociedad de Cirugía de Bogotá and at Hospital Infantil Universitario de San José de Bogotá, Bogotá, Colombia, between January 2015 and December of 2023. We included patients > 18 years old who were surgically treated through maximal safe resection or an intentional biopsy and had a histologically confirmed diffuse glioma, data for retrieval was available through medical records. Only patients diagnosed with oligodendroglioma (Grade II and III), astrocytomas (grade II and III) and GBM were included (**Figure 1**). Patients in which the similarities to neuroglial cells were not identified and were reported as grade II low-grade glioma (LGG), grade II or III oligoastrocytomas, and grade III high-grade glioma (HGG), were classified as ‘glioma, *not otherwise* sp*ecified’* (glioma, NOS) grade II and III, and were also included. Patients with data not available were excluded, accordingly. Patients diagnosed with Grade I gliomas (e.g. pilocytic astrocytomas) were excluded, as they are considered different entities and this study focused only on diffuse gliomas (**Figure 2**), as already mentioned.

**Figure 3:**
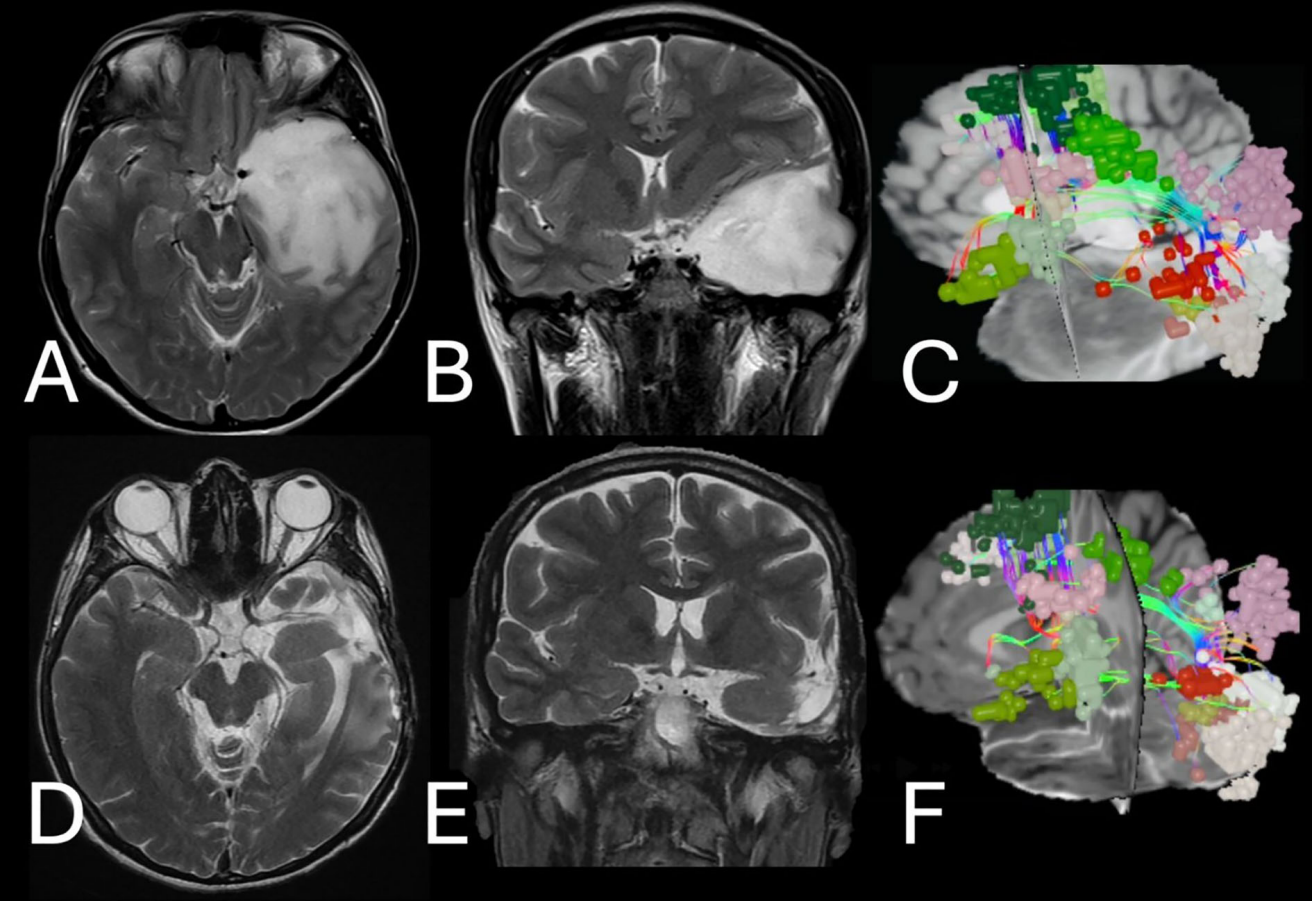
**⁠**Illustrative case – left temporal grade II astrocytoma. **(A, B)** Pre- and **(D, E)** postoperative T2 images demonstrate the resection of a large left temporal mass concerning the language area. **(C)** Pre- and **(F)** postoperative reconstruction of the language system including neural networks (small color spheres) and language tracts (color fibers). Postoperative reconstruction demonstrates the preservation of all language areas after awake craniotomy while preserving language function.

Electronic records of both institutions were reviewed. Demographic and clinical data were collected including histopathological diagnosis, immunohistochemical markers, radiological findings, clinical symptoms, tumor location, pre- and postoperative Karnofsky Performance Score (KPS), the extent of resection (EOR), complications, and OS. Gliomas were classified using the WHOCNS4 (5) and were grouped accordingly. EOR was classified into gross-total resection (GTR) (complete radiological resection), near-total resection (NTR) (>90%), subtotal resection (STR) (<90%), and intentional biopsy. EOR was calculated for GBM considering resection of the enhancing area of the tumor, while for grade II and III gliomas, it was calculated based on the hyperintense area on T2/FLAIR. Maximal safe resection was aimed for each individual case, through strategies and techniques such as tractography and reconstruction of eloquent structures for awake craniotomy, as seen in **Figure 3** which depicts the resection of a left temporal grade II astrocytoma in one of the patients. Awake craniotomy was used only for tumors related to language areas. Otherwise, the rest of the cases were performed in an asleep manner. Our protocols have been previously reported elsewhere (16–20).

For OS analysis, the national statistical system Departamento Administrativo Nacional de Estadística (DANE) and the National Registry of Civil Status of Colombia (Registraduría Nacional del Estado Civil) registries were consulted. This study was approved by the Institutional Review Board and the Ethics Board of the Fundación Universitaria de Ciencias de la Salud under approval ID number I–0328-22. This study was performed following the principles outlined in the Declaration of Helsinki.

### 2.1 Statistical analysis

Variables were grouped into numerical, binary categorical, and nominal. The means were analyzed with the Kruskal-Wallis test. The t-student test and the Chi-square test were used accordingly. OS was assessed using the Kaplan-Meier test. The *RStudio v06.1^®^*, using packages including *survival* and *survminer*, was used to make a statistical analysis for p-value calculations. P-value <0.05 was considered statistically significant.

## 3 Results

A total of 272 patients with confirmed diffuse gliomas were included in the study. 38.20% were classified as glioma, NOS, 36.00% GBM, 14.70% oligodendroglioma, and 12,1% astrocytomas. 49.10% of patients were female, and the average age was 48.80 ± 21.00 years. The most common location for all tumors was the frontal lobe (37.50%), followed by the insula (16.50%) (**Table 1**). The frontal lobe was the most common location for GBMs (38.95%) and oligodendrogliomas (47.50%), while astrocytomas were more frequent in the insula (27.27%). 87.50% of the cases were supratentorial, and less than 2.00% had a bilateral compromise. Most of the patients were right-handed (92.28%), and in 49.63% of the patients, the tumor was in the right hemisphere. There were no statistically significant differences between pre- and postoperative KPS scores in all groups.

**Table 1:** Clinical and demographic data.

Variable	Astrocytoma (n = 33)	Glioma/NOS (n= 104)	GBM (n = 98)	Oligodendroglioma (n=40)	Total (n =272)	P-value
*Sex* (number [%])						
Male	54.55%	35.58%	51.63%	45.00%	44.85%	0.39
Female	45.45%	64.42%	48.37%	55.00%	55.15%	0.39
*Age* (mean ± SD)	39.3 ± 20.36	43.67 ± 22.77	59.24 ± 16.33	44.83 ± 16.00	48.84 ± 20.95	0.39
*Pathology* (number [%])						
LGG	51.52%	48.00%	N/A	68.00%	34.60%	0.18
HGG	48.48%	48.00%	N/A	33.00%	29.00%	0.17
*Location* (number [%])						
Frontal	12.12%	33.65%	38.95%	47.50%	37.50%	0.13
Frontoparietal	6.06%	5.77%	5.26%	17.50%	7.30%	0.09
Parietal	6.06%	8.65%	13.68%	17.50%	11.40%	0.13
Insular	27.27%	14.42%	14.74%	17.50%	16.50%	0.13
Basal Ganglia	9.09%	6.73%	2.11%	0.00%	4.40%	0.39
Temporal	3.03%	14.42%	18.95%	0.00%	4.50%	0.39
Posterior fossa	9.09%	7.69%	2.11%	0.00%	12.40%	0.18
Bilateral	6.06%	4.81%	3.16%	0.00%	1.40%	0.39
Occipital	0.00%	3.85%	0.00%	0.00%	4.60%	1.00
*Patients’ laterality* (number [%])						
Right sided	6.06%	7.69%	8.47%	7.50%	92.28%	0.39
Left sided	93.94%	92.31%	90.53%	92.50%	7.72%	0.13
*Hemisphere compromised* (number [%])						
Right	54.50%	48.10%	49.50%	50.00%	49.63%	0.39
Left	33.30%	40.40%	49.50%	50,00%	44.12%	0.39
Midline or infratentorial	3.00%	10.60%	1.10%	0.00%	4.78%	0.13
Bilateral	9.10%	1.00%	0.00%	0.00%	1.47%	0.39
*Presenting symptom* (number [%])						
Seizures	33.33%	39.42%	34.74%	57.50%	39.71%	0.39
Motor deficit	30.30%	45.19%	50.53%	30.00%	42.65%	0.13
Language	12.12%	25.00%	31.58%	15.00%	24.63%	0.39
Sensorial deficit	45.45%	48.08%	50.53%	40.00%	47.79%	0.39
Behavior impairment	30.30%	28.85%	34.74%	25.00%	30.51%	0.39
Other	9.09%	22.12%	9.47%	10.00%	14.71%	0.39
*Follow-up in months* (mean ± SD)	36.32 ± 18.34	1.70 ± 18.85	11.97 ± 10.46	27.39 ± 14.28	11.75 ± 15.98	0.39
*Extent of resection* (number [%])						
GTR	12.12%	12.50%	9.47%	15.00%	11.45%	0.39
NTR	33.33%	38.46%	38.95%	52.50%	40.10%	0.39
STR	36.36%	34.62%	45.26%	22.50%	37.00%	0.39
Biopsy	18.18%	14.42%	6.32%	10.00%	11.45%	0.18
*New onset postoperative seizures* (number [%])	15.15%	17.30%	18.95%	20.00%	18.01%	0.39
*New onset language deficit* (number [%])	15.15%	23.10%	22.11%	5.00%	19.49%	0.39
*New onset motor deficit* (number [%])	15.15%	37.50%	34.74%	20.00%	31.25%	0.39

* In those cases where the tumor grade was not reported, they were not included in percentages of LGG and HGG.

*⁑* The LGG group includes only grade II gliomas and the HGG group only grade III gliomas.

**Figure 4:**
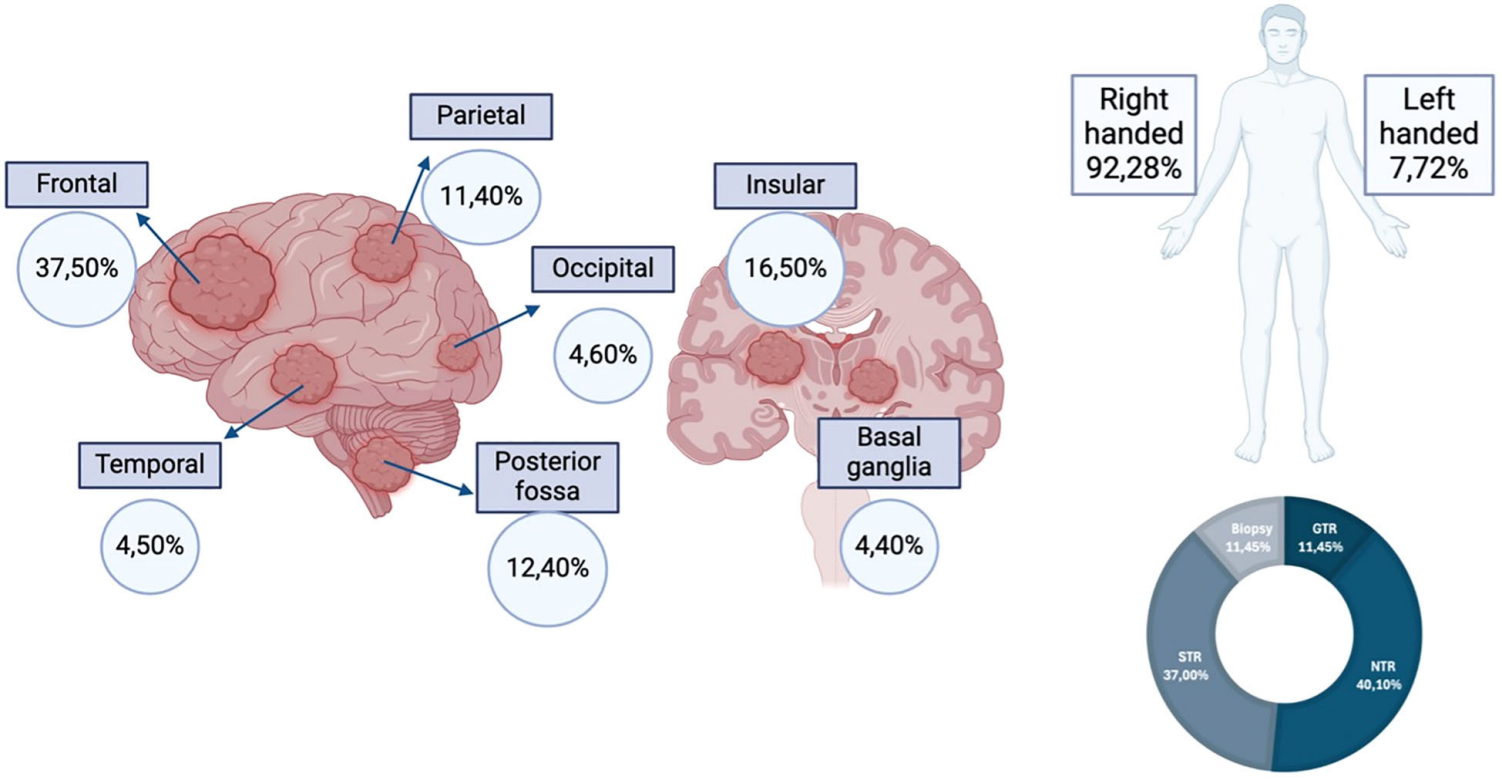
Tumor distribution by location, patient laterality and extent of resection. *GTR, gross-total resection; STR, subtotal resection; NTR, near-total resection.* Created using www.biorender.com.

The average follow-up was 11.8 ± 16.0 months. However, the middle-term follow-up for OS analysis was carried out using national registries, although they were not followed up in our institutions afterward. NTR was achieved in 40.10% of the patients, while STR was achieved in 37.00%, GTR in 11.45%, and intentional biopsy in 11.45%. 18.01% of the patients had new-onset postoperative seizures, and all of them were treated successfully with anticonvulsants alone. 19.49% of the patients had new-onset language deficits, however, only 5.00% were persistent 3 months after resection. 31.25% of the patients had new-onset motor deficits, even though only 3.00% persisted after the 3-month follow-up. Data on tumor location, patient laterality, and extent of resection are summarized in **Figure 4**.

No statistically significant differences were found in the positivity of immunochemistry markers between oligodendroglioma, glioma, NOS, astrocytomas, and GBM (**Table 2**). The average Ki-67 proliferation index was 11.00% for oligodendroglioma, 14.06% for glioma NOS, 10.21% for astrocytoma, and 34.56% for GBM (p = 0.063).

**Table 2:** Immunochemistry markers profiles.

Variable	Astrocytoma (n = 33)	Glioma/NOS (n= 104)	GBM (n = 98)	Oligodendroglioma (n=40)	Total (n =272)	P value
P53 (+)	65.00%	68.66%	82.26%	79.17%	74.14%	0,063
SIPNASIS (+)	64.71%	67.86%	55.32%	50.00%	61.70%	0,063
CK (+)	50.00%	57.69%	6.32%	60.00%	42.37%	0,063
CD34 (+)	80.00%	84.13%	84.75%	72.00%	81.55%	0,063
CAM5.2 (+)	N/A	66.67%	25.00%	100.00%	42.11%	0.088
S100 (+)	84.62%	90.70%	86.96%	80.00%	87.18%	0.057
EGFR (+)	77.78%	81.48%	85.71%	100.00%	84.88%	0.057
GFAP (+)	100.00%	97.37%	98.63%	100.00%	98.12%	0.063
IDH(+)	63.64%	52.00%	33.33%	61.54%	48.37%	0.063
OLIG2 (+)	93.75%	84.31%	78.26%	96.55%	85.31%	0.057
INI-1 (+)	N/A	47.62%	80.00%	N/A	73,33%	0.181
SOX 10 (+)	80.00%	71.43%	63.64%	60,00%	68,06%	0.058
PHH3 (+)	33.33%	70.59%	72.73%	53.85%	41.51%	0.057
EMA (+)	33.33%	57.14%	58.06%	33.33%	53.23%	0.058
CD15/45/3/20/68 (+)	50.00%	52.63%	50.00%	12.50%	45.90%	0.058

### 3.1 Survival analysis

**Figure 5:**
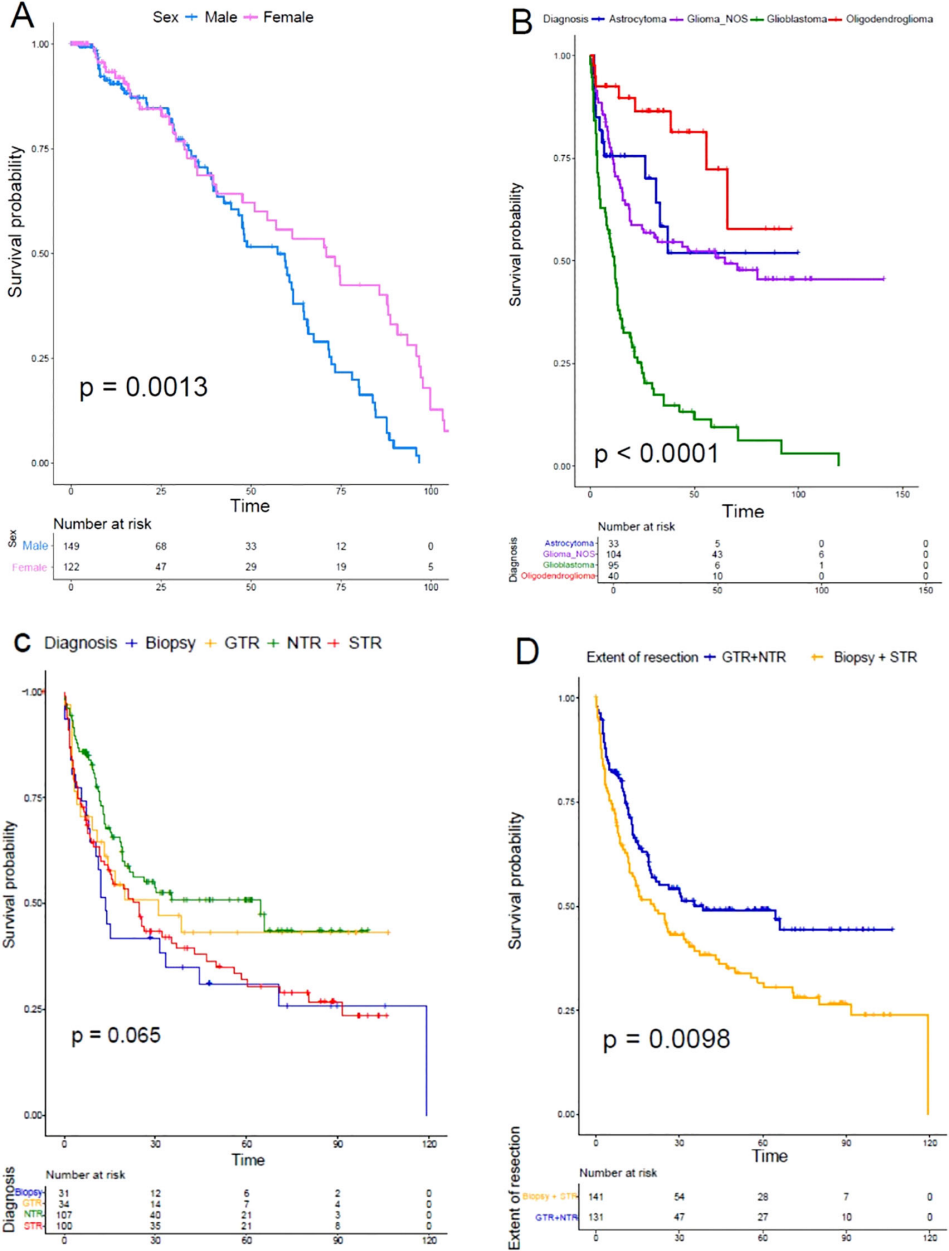
Kaplan-Meier survival analysis. **(A)** Differences in overall survival of females (pink) versus males (blue) (p = 0,0013). **(B)** Differences in overall survival according to histopathological diagnosis (GBM in green, Glioma, NOS in purple, astrocytoma in blue, and oligodendroglioma in red) (p < 0,0001). **(C)** Differences in overall survival according to the extent of resection. (GTR in yellow, NTR in green, STR in red, and biopsy in blue) (p = 0,065). **(D)** Differences in overall survival between GTR + NTR versus STR + intentional biopsy (p = 0,0098). No computational methods, such as artificial intelligence-based feature extraction, were used for this analysis.

OS was higher in females (p = 0.0013) (**Figure 5**). As expected, OS was higher in the following order: oligodendroglioma, astrocytoma, glioma, NOS, and GBM. The average OS for each histological type was as follows: oligodendroglioma: 36.94 months, astrocytoma: 26.78 months, glioma NOS: 42.73 months, and GBM: 16.54 months (p < 0.0001). The 2-year OS for GBM was 21%, the 5-year OS for glioma, NOS 38%, the 5-year OS for astrocytoma 15%, and the range of 2–5 years, and 8 years for oligodendroglioma were 20% and 5%, respectively (p < 0.0001). Differences in OS according to EOR were not statistically significant: gross total resection (GTR): 30.70 months, near-total resection (NTR): 30.72 months, subtotal resection (STR): 30.79 months, and intentional biopsy: 29.93 months (p = 0.065); however, it was statistically significant when performing analysis between GTR + NTR (30,71 months) versus STR + biopsy (30,37 months) (p = 0.0098). There were differences in OS according to EOR (GTR vs NTR vs ST vs biopsy) in all histological subtypes, however, it was statistically significant only for astrocytoma (GTR 24,25: months, NTR: 28,81 months, biopsy 18,16 months, STR: 30,25 months) (p = 0.038). There were no statistical differences when performing STR versus intentional biopsy (STR: 30,79 months, biopsy: 30,04 months) (p = 0.74).

## 4 Discussion

We present the largest study of diffuse gliomas in Colombia to date. Colombia’s population genome has shown the highest levels of average three-way admixture contributions from ancestral populations (60% European, 29% Native American, and 11% African) as well as the greatest extent of geographical variation in genetic ancestry, compared to other countries like Mexico, Ecuador, Dominican Republic, and Puerto Rico (21). This is extremely relevant, as Walsh et al. have demonstrated that glioma incidence and outcomes differ in association with the geographic origins of Hispanic communities when comparing Mexican/Central American origin versus those of Caribbean origin (22). Although the Colombian population has been classified as a single ethnic group (Hispanic), the cultural, socioeconomic, and genetic diversity is large. Our institutions are in Bogotá, where patients from all over the country are referred, especially from departments in the country’s center (Cundinamarca, Boyacá, Tolima, and Huila). We have included clinical and histological diagnoses of tumors, adding relevant information for further research. Even though all cases were classified only based on histological findings, this posed a limitation in accurately classifying many tumors. Despite this, we have included all possible information to analyze the OS in all groups.

Most gliomas are more common in males (23), even though, in our study, we found an increased number in females (55.15%). As mentioned before, GBM is the most frequent glioma. We also found that the most common confirmed subtype was GBM (98 cases), however, many tumors were classified as glioma, NOS, likely related to the lack of molecular information, which could help to further classify them into oligodendroglioma or astrocytoma and guide targeted therapy.

Regarding new-onset symptoms at presentation, patients with low-grade astrocytomas and oligodendrogliomas present with seizures in ~60%–88% of cases (24). In our study, 33.33% of patients with astrocytoma and 57.50% of patients with oligodendroglioma presented with seizures. As well as reported in other studies (25, 26), in our study the most frequent location for all tumor subtypes was the frontal lobe (37.50%).

Many glioma studies have been carried out in Latin American countries, mainly in México (27–30), Brazil (31–33), Argentina (34, 35), and Chile (36). In all of these countries, the caseload of patients, given the presence of large cancer centers, represents a significant difference compared to smaller countries of limited economic resources like Colombia. Furthermore, the integration of molecular markers like the 1p19q codeletion and telomerase reverse transcriptase (TERT), among others included in the WHOCNS5 (7) has increased the disparity between diagnosis and targeted therapy of diffuse gliomas in LMICs compared to higher-income countries (HICs). This could potentially be assessed through different artificial intelligence (AI) methods, especially using histopathological protocols fed with molecular information from HIC studies, while using machine learning and deep learning (37, 38). Even almost providing real-time information when histopathological analysis is available (39). This information can be used from freely available datasets like those from The Cancer Genome Atlas (TCGA) (40) and could not only provide diagnostic information but also prognosis in terms of OS (41). This applies not only to adjuvant therapy but also to surgical technologies used in high-income countries, like the use of new magnification equipment like three-dimensional exoscopes (42), the use of electrocorticography (ECoG) grids (43), intraoperative handheld endomicroscopy for ex vivo glioma diagnosis and *in vivo* roving scan in navigation (44), and treatments like tumor treating fields (45).

Regarding tumoral behavior, in this study, we found a similar behavior of gliomas in comparison to data from other countries in our region and worldwide. GBM remains the most aggressive and frequent glioma. We achieved a GTR/NTR in 51.55% of patients, improving OS when compared to those patients treated with STR or intentional biopsy (p = 0.0098). No statistical differences between the positivity of immunochemistry markers among the different types of tumors were found, making it necessary to perform further studies on the behavior of molecular markers between them.

Even though access to molecular marker tests remains scarce in our country, given the high costs associated with kits to perform specific molecular tests, including fluorescence *in situ* hybridization, quantitative real-time polymerase chain reaction, or next-generation sequencing. To date, few institutions can afford to perform these tests, and only after 2022 did the Colombian healthcare system include them in the list of available tests covered by healthcare insurance companies. The next steps of diffuse glioma research in our population include the reclassification of tumors according to the WHOCNS5 and the sequencing of tumor samples (RNAseq, exosome, etc.) to elucidate the relation between tumor features and genetic ancestry.

Finally, when observing EOR concerning OS, no statistically significant differences were found, yet when comparing GTR + NTR versus STR + biopsy, there was increased OS for GTR + NTR, which was statistically significant. Several studies have described the maximal safe tumor resection as the standard of care in patients with diffuse gliomas and cases such as for the multimodal treatment of glioblastoma along with chemoradiotherapy (Stupp’s regimen) (46, 47). It has been widely discussed, but an extensive EOR has been increasingly associated with improved overall survival and progression-free survival due to factors such as reduced tumor volume near critical brain areas and enhanced response to adjuvant therapy (46, 48). Nonetheless, factors such as inconsistent definition and quantification of the EOR in trials have limited the value of the interpretation of the oncologic effects of the EOR in glioma surgery, and further study is required to adequately quantify EOR (48, 49).

### 4.1 Limitations

Amongst the study limitations, the diagnosis of diffuse gliomas in our study relied on the accuracy of the histopathological analysis. Classification of some gliomas could be therefore erroneous. Moreover, the molecular profiling of tumors was not evaluated. We encourage neuroscientists to continue developing research on molecular analysis as the information is still scarce and limits the information for clinical use aiming to improve patient care. While some patients had better OS when achieving NTR compared to some who underwent GTR, this could be explained by lack of accuracy of molecular diagnosis and other factors that could have altered this outcome, including tumor location and size. Finally, ethnicities and genetic ancestries were not evaluated, therefore genetic and associated molecular features were not examined yet describing these could provide valuable information for the collected data obtained from a widely racially heterogeneous population.

## 5 Conclusions

We describe the clinical, histopathological, and demographic features of diffuse glioma patients treated in two reference centers in Colombia. Clinical findings and OS trends are similar to those reported worldwide, however, further molecular and genetic analysis is required for adequate diagnosis and classification.

## Data Availability

The raw data supporting the conclusions of this article will be made available by the authors, without undue reservation.
